# ProDiVis: a method to normalize fluorescence signal localization in 3D specimens

**DOI:** 10.3389/fcell.2024.1420161

**Published:** 2024-09-23

**Authors:** Kyle T. Nguyen, Alexandre R. Sathler, Alvaro G. Estevez, Isabelle E. Logan, Maria Clara Franco

**Affiliations:** ^1^ Department of Biochemistry and Biophysics, Oregon State University, Corvallis, OR, United States; ^2^ Herbert Wertheim College of Medicine, Florida International University, Port St. Lucie, FL, United States; ^3^ Center for Translational Science, Florida International University, Port St. Lucie, FL, United States

**Keywords:** 3D specimen, heatmap, confocal microscopy, fluorescence microscopy, protein distribution, signal normalization, imaging, image analysis

## Abstract

A common problem in confocal microscopy is the decrease in intensity of excitation light and emission signal from fluorophores as they travel through 3D specimens, resulting in decreased signal detected as a function of depth. Here, we report a visualization program compatible with widely used fluorophores in cell biology to facilitate image interpretation of differential protein disposition in 3D specimens. Glioblastoma cell clusters were fluorescently labeled for mitochondrial complex I (COXI), P2X7 receptor (P2X7R), β-Actin, Ki-67, and DAPI. Each cell cluster was imaged using a laser scanning confocal microscope. We observed up to ∼70% loss in fluorescence signal across the depth in Z-stacks. This progressive underrepresentation of fluorescence intensity as the focal plane deepens hinders an accurate representation of signal location within a 3D structure. To address these challenges, we developed ProDiVis: a program that adjusts apparent fluorescent signals by normalizing one fluorescent signal to a reference signal at each focal plane. ProDiVis serves as a free and accessible, unbiased visualization tool to use in conjunction with fluorescence microscopy images and imaging software.

## Introduction

Confocal microscopy is a robust and widely used tool for imaging and analysis of cells and tissue samples in biological research (Justyna, 2017). Optical sectioning routinely provides a way to visualize internal structures of biological material. A primary advantage is the ability to provide depth information without physically cutting a specimen. However, there are limitations to optical sectioning while imaging thick specimens, including fluorescence intensity loss as a function of imaging depth. Many common fluorophores emit light that can only penetrate a limited distance through biological material (Kobat et al., 2011; Benninger and Piston, 2013). This is a contributor to the fundamental depth limit, caused by several physical properties such as light scattering and absorption (Benninger and Piston, 2013).

We developed a computational method to proportionally compare pixel values across the depth of 3D specimens, accounting for the decrease in fluorescence intensity we and others have previously observed (Helmchen and Denk, 2005; Kobat et al., 2011; Brenna et al., 2022; Čapek et al., 2006). While there are existing programs available for such an endeavor, to the best of our knowledge, none account for loss of signal by normalizing against a housekeeping signal, and they are expensive and/or require a high level of technical expertise to use. For example, the popular commercial software Imaris (RRID:SCR_007370) is costly, and its use requires considerable experience. On the other hand, the free, open-source software package ImageJ/Fiji (RRID:SCR_003070) is versatile, but complex for novice users (Schneider et al., 2012; Brenna et al., 2022).

To maximize accuracy of signal distribution in a 3D specimen, critical when studying macromolecules that are not homogenously distributed within a 3D structure, we developed ProDiVis, an accessible Z-stack validation suite written in the python programming language (RRID:SCR_008394), designed to be user-friendly. ProDiVis allows users to analyze Z-stacks quickly and efficiently. Our method takes Z-Stack outputs acquired from bioformat files (CARL ZEISS .czi or Leica .lif) and generates a heatmap of differentially localized protein(s) normalized to a user-selected fluorescent housekeeping signal. Heatmaps can be generated from any fluorescent Z-stack and serve as an unbiased visualization tool. In addition to heatmaps, ProDiVis outputs a normalized Z-stack that can be readily used with concurrent microscopy images. To facilitate image analysis, ProDiVis provides built-in tools to show useful sample information such as depth-dependent signal intensity loss and pixel value distribution pre- and post-normalization.

ProDiVis is run entirely in a Jupyter notebook (RRID:SCR_018315) that requires minimal user input. ProDiVis analyzes and normalizes one Z-stack at a time with two fluorescent channels. The first channel is the user-selected signal of interest (SOI). The second channel corresponds to the user-selected normalization signal (NS). When evaluating biological features in model organisms, imaging each sample presents its own unique challenges. Therefore, choosing a suitable NS signal is at the discretion of the user. When using ProDiVis, each Z-stack must originate from the same sample and should feature optical sections of identical thickness and resolution. Normalization by ProDiVis begins with histogram thresholding, which segments an image by setting a range of pixel values to be considered for analysis. This selects the object of interest within the image, where ProDiVis excludes any pixel value(s) outside of the user-defined boundaries.

When using ProDiVis, users must consider the following: 1) fluorescence signal lost by the NS and SOI are proportional to each other. For example, a probe that photobleaches rapidly for the NS while the SOI exhibits greater stability would reduce normalization quality. 2) The chosen NS has uniform distribution throughout sample depth, or a distribution similar to that expected for the SOI, which eliminates underrepresented information that may be lost. This may be a fluorescently labeled housekeeping protein or a fluorescent DNA stain such as 4′,6-Diamidino-2-phenylindole (DAPI), depending on the context. 3) Histogram thresholding is sufficient to distinguish signal from background. Image segmentation by histogram thresholding a widely used method to separate signal from background (Tobias and Seara 2002; Li et al., 2020). ProDiVis assumes that anything set outside the bounds of the user-defined threshold is background or an experimental artifact.

**FIGURE 1:**
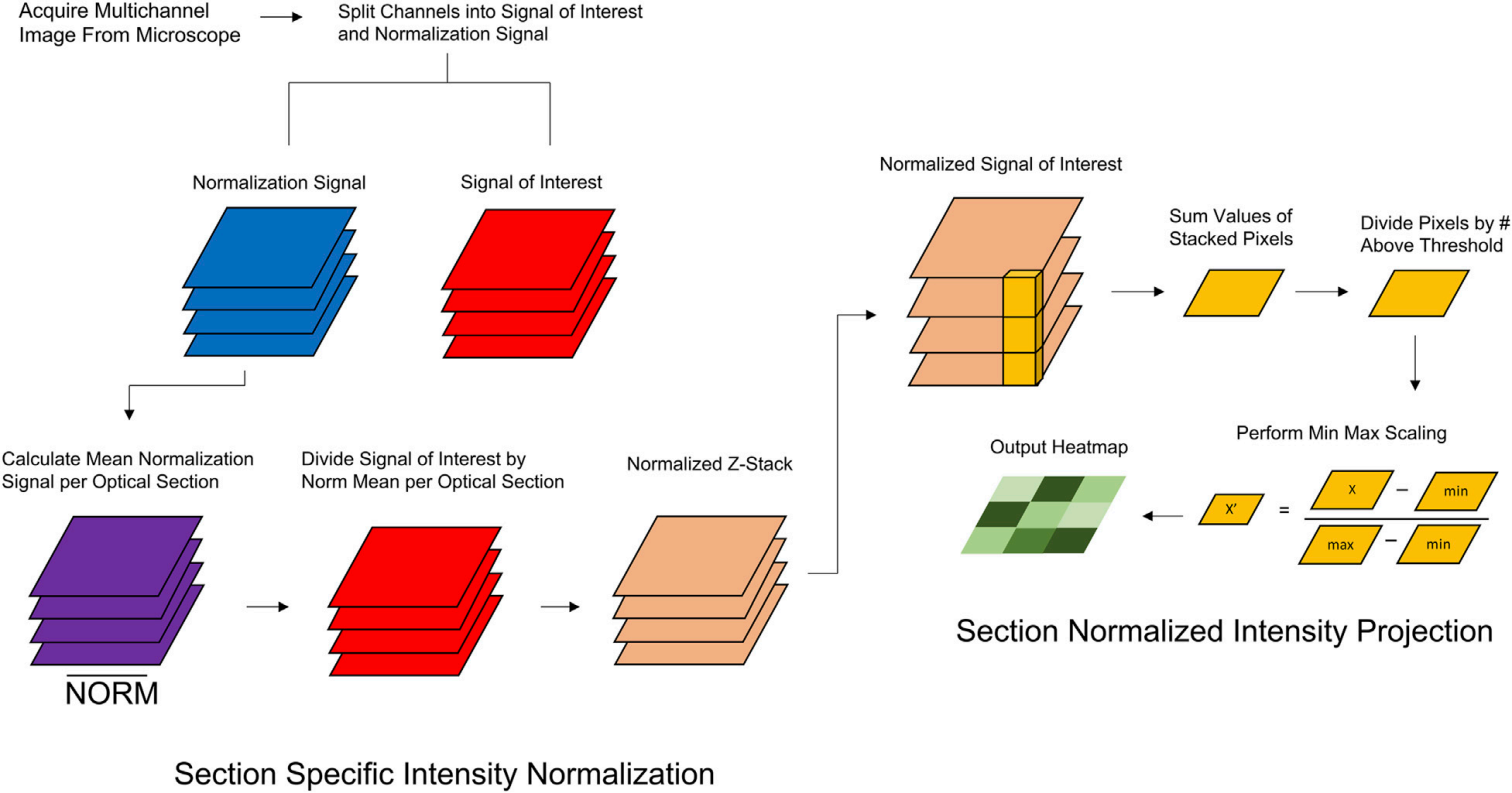
Schematic Overview of SsIN and SNIP. Prior to image analysis, ProDiVis requires a multichannel image taken from a fluorescent microscope. The normalization signal and signal of interest must be separated individually into separate directories. **(A)** The average NS at each optical section is stored. The SOI signal is then divided by each corresponding NS. **(B)** Using the normalized images produced by SsIN, SNIP produces an orthogonal projection of the average pixel values across the x, y, and z dimensions which are then min/max scaled and displayed in a heatmap.

To preserve the biological features of raw image data while accounting for the decrease in fluorescence as a function of depth, we developed Section-Specific Intensity Normalization (SsIN). SsIN normalizes fluorescence signal distribution in Z-stacks acquired with multi-color imaging (Figure 1). SsIN first determines the non-zero mean of the NS at each optical section of a Z-stack. Then, ProDiVis performs a pixel-wise division of SOI signal intensity using the NS mean at the corresponding focal depth to create a new, normalized Z-stack. Applying SsIN provides multiple advantages to image analysis in a biologically relevant context: 1) determination of SOI distinct spatial distribution within a 3D structure by minimizing methodological artifacts such as light attenuation or other factors hindering proper signal detection, 2) creation of a normalized Z-stack that can be displayed in color images similar to confocal software, 3) can be used in conjunction with another parameter in ProDiVis, which creates a multidimensional heatmap that shows the areas in a sample where each SOI is predominantly localized.

In addition to normalizing fluorescence intensity with SsIN, we developed an intensity projection function in ProDiVis. Section-Normalized Intensity Projection (SNIP) provides an additional way to visualize normalized Z-stacks produced by ProDiVis by displaying the average intensity of x, y, and z dimensions simultaneously (Figure 1). SNIP has multiple functions: 1) generates a heatmap of protein localization from any unprocessed Z-stack, 2) can be used coupled to SsIN to create a heatmap using the normalized Z-stack produced by SsIN. SNIP also produces orthogonal projections, aiding in the visualization of a particular SOI. Therefore, used in combination with SsIN, SNIP allows the user to gain insight on the biological features of protein distribution in a 3D sample.

SNIP begins by computing the mean of parallel grayscale values at each x-y coordinate in a Z-stack to produce an x-y matrix, identical to how mean intensity projections are calculated (Ohira et al., 2016). To utilize the entire visual range in the output image, min-max scaling was applied: the smallest values in the image are scaled to 0 while the largest values are scaled to 255 (8-bit scale). SNIP can therefore highlight local regions of high signal intensity that may be of particular interest.

In short, ProDiVis is a comprehensive Z-stack validation suite with several different features. ProDiVis generates histograms of pixel values for the NS and SOI; users can visualize data distribution before and after normalization. ProDiVis also provides a graphical representation of depth loss for both NS and SOI. Normalization and image rescaling features are provided, and a built-in graphical user interface allows for visualization of normalized images. The built-in heatmap feature is particularly useful for both highlighting signal distribution throughout the specimen and visualizing the impact of normalization on SOI intensity. Overall, ProDiVis is a simple yet effective program that provides biologists with a powerful tool for Z-stack analysis and validation, while preserving the biological features of the sample.

## Materials and methods

### Cell culture and immunostaining

U87-MG cells were obtained from American Type Culture Collection: U87-MG (ATCC HTB-14, RRID:CVCL_0022). Cells were maintained at 37°C and 5% CO_2_ with humidity on 10 cm dishes (Fisher Scientific Cat. No. FB012924) in DMEM with 4.5 g/L glucose and glutamine without sodium pyruvate (Corning Cat. No. 10-017-CV) supplemented with 10% FBS (ScienCell Cat. No. 0500) and 1% penicillin/streptomycin (Gibco Cat No 15140). U87-MG cells were seeded at a density of 50,000 cells per chamber in an 8 chamber Permanox^®^ slide (Thermo Fisher Scientific Cat. No. 177445) and allowed to form cell clusters for 48–72 h. Cells were prefixed in each chamber with 200μl of cell culture media and 200 μl of 4% paraformaldehyde, 0.2% glutaraldehyde solution for 10 min on ice. Following prefixation, cells were washed with PBS 3x for 5 min and incubated with 200μl of fixation buffer (4% paraformaldehyde, 0.2% glutaraldehyde) for 30 min at room temperature. Cells were washed again with PBS 3x for 5 min and incubated with 50 mM glycine +0.1% Triton-X for 30 min. Cells were then blocked with blocking solution (10% goat serum (Gibco Cat. No. 16210-064) + 0.2% Triton-X) for 1 h at room temperature.

Primary antibodies were diluted into blocking solution and were incubated in each chamber overnight at 4°C, followed by 3 washes with PBS +0.1% Triton-X for 15 min the following day. Primary antibodies are as follows: COXI (Thermo Fisher Scientific Cat. No. 459100, RRID:AB_2532223) at 1:250; P2X7R (Alomone Labs Cat. No. APR-004, RRID:AB_2040068) at 1:100; Actin (Cell Signaling Cat. No. 3700s -also 3700P, 3700S- RRID:AB_2242334) at 1:5,000. Ki-67 (Abcam Cat. No. AB16667, RRID:AB_302459) at 1:250. This step was identical for secondary antibody incubation using Goat anti-Rabbit IgG Alexa Fluor 488™ (Thermo Fisher Scientific Cat. No. A-11008 -also A11008- RRID:AB_143165) at 1:2000, and Goat anti-Mouse IgG Alexa Fluor 594™ (Thermo Fisher Scientific Cat. No. A-11005, RRID:AB_2534073) at 1:2000. Prior to mounting the coverslip, cell clusters were incubated with DAPI (Thermo Fisher Scientific Cat. No. D1306) for 45 min at room temperature in PBS and washed twice with PBS +0.1% Triton-X for 15 min. The resulting slide was mounted with a #1.5 cover slip (Thermo Fisher Scientific Cat. No. 152250) using Prolong™ Gold Antifade Mountant (Thermo Fisher Scientific Cat. No. P10144).

### Confocal imaging

Mounting Medium was allowed to cure for at least 24 Hours. Each cell cluster was imaged on a Zeiss LSM 780 NLO confocal microscope using a ×40 oil objective and the pinhole was set to 1 AU. For image acquisition, cell clusters were located and optically sectioned to adjust laser power in each channel, ensuring no pixels would become saturated. Laser power was adjusted separately for each cell cluster. To scan and image cell clusters, we determined the optical section at the top and bottom of cell clusters. Those focal planes were saved in ZEN software (RRID:SCR_018163) and line scanning began at the top of cell clusters moving down the specimen. Images were acquired either as an 8-bit dynamic or 16-bit dynamic range and saved as a. czi file.

### Visualizing depth loss and normalization using ProDiVis

Using ZEN software or Imagej/Fiji, images were split into their corresponding color channels (red, green, or blue), then split by the *Z*-axis and exported in .tiff format to a new directory as an image sequence, where each image in its final folder corresponds to a single color at a single depth in the specimen.

Within the ProDiVis Jupyter notebook interface, the file path to the NS folder and SOI folder are assigned by the user. Before analysis, the user is also required to assign a pixel value threshold range to consider for analysis. Any pixel value below or above the bounds of the threshold will be excluded. This is especially useful when analyzing images with known experimental artifacts. For example, antibody precipitation can occur in one or more optical planes. Antibody precipitation usually contains saturated punctate areas, and those pixels may skew interpretation in the downstream analysis.

After these initial parameters, ProDiVis uses the OpenCV (RRID:SCR_015526) python library (Bradski, 2000) to import images while preserving bit-depth. If input images are in grey-scale, they are processed as-is. If input images are multichannel, they are converted to grayscale using OpenCV’s BGR2GRAY lookup table. Any pixel values outside of the threshold range set by the user, including any zero value pixels are not included in the mean calculation. The image means are calculated for the NS and SOI separately and the maximum mean value for each respective signal is determined. Depth loss plots are then produced as the mean pixel value per optical section as a percentage of the maximum average pixel value of the entire Z-stack.

An image histogram helps a user visualize the range and abundance of pixel values that are being represented, although this can be overwhelming when viewing tens or hundreds of individual histograms for thick Z-stacks. We therefore implemented a parameter in ProDiVis that condenses each histogram in a Z-stack into a single 3-dimensional plot. The *x*-axis represents histogram bin, while the *y*-axis represents optical section number, and the color represents count. To plot histograms of each image in a respective Z-stack, images were analyzed using the same method above. ProDiVis reads every image in a Z-Stack and creates a histogram of each image using the NumPy (RRID:SCR_008633) library. Histograms are appended together in a list and represented as a heatmap using a log scale color bar.

For the purpose of normalization, we applied histogram threshold segmentation only to the SOI and not the NS. We found that much of the depth loss information was stored in low (0–10) pixel values of the selected NS. Once each mean of the NS was calculated, it was aligned with each optical section of the SOI at its corresponding index. Pixel by pixel, the SOI value was divided by the average NS value, generating a normalized image. As each SOI image is normalized, it is output into a new directory with its native resolution and bit-depth. Normalized images can then be assigned any color in image analysis software as required by the user(s).

Analysis using the 8-bit dynamic range may present drawbacks, due to the smaller range of possible values to represent pixel or fluorescence signal intensity. This becomes more apparent in ProDiVis since the program applies division to each individual pixel. Dividing by a number in an 8-bit image (256 possible values) impacts the values more than dividing that same number in a 16-bit image (65,536 possible values), since integers in the 8-bit image represent a larger percent of the total dynamic range. To meet the user’s need, ProDiVis can process images in any dynamic range.

### Heatmap generation

Orthogonal projections are commonly used in many areas of microscopy, including neuroscience, cell biology, and developmental biology. We created a projection method similar to methods that are currently used. SNIP therefore combines an average intensity projection with an orthogonal projection. As each image is analyzed, SNIP stores the pixel values in a multidimensional matrix, whose size is dependent on the resolution of the image. Across the x, y, and z planes within the matrix, the mean of pixel values superimposed on each other will be calculated. This results in numbers which each represent the x, y, and z dimensions of the sample which are then min/max scaled and displayed in a heatmap at the same resolution as the original input images.

### Scaling images

While SsIN effectively accounts for depth loss, dividing by a normalization mean can lead to lower pixel values in the normalized Z-stack. An optional post-processing brightness and contrast adjustment was implemented *via* a basic linear transform. A graphical user interface integrated into ProDiVis allows users to scroll through the output image stack to select a reference focal plane to scale by. Images across the Z-stack are multiplied by a scale factor (α) and adjusted by an offset factor (β) (Szeliski, 2021). First, α is calculated by dividing the maximum intensity of an 8-bit image by the intensity range set by the user when viewing each image. Next, β is calculated by multiplying the user-selected minimum intensity by α. This maps the user-selected minimum intensity to the zero percentile and maps the user-selected maximum intensity to the 100th percentile. The adjusted stack is then saved to the user’s filesystem.

## Results

### Fluorescence intensity decreases with imaging depth

**FIGURE 2:**
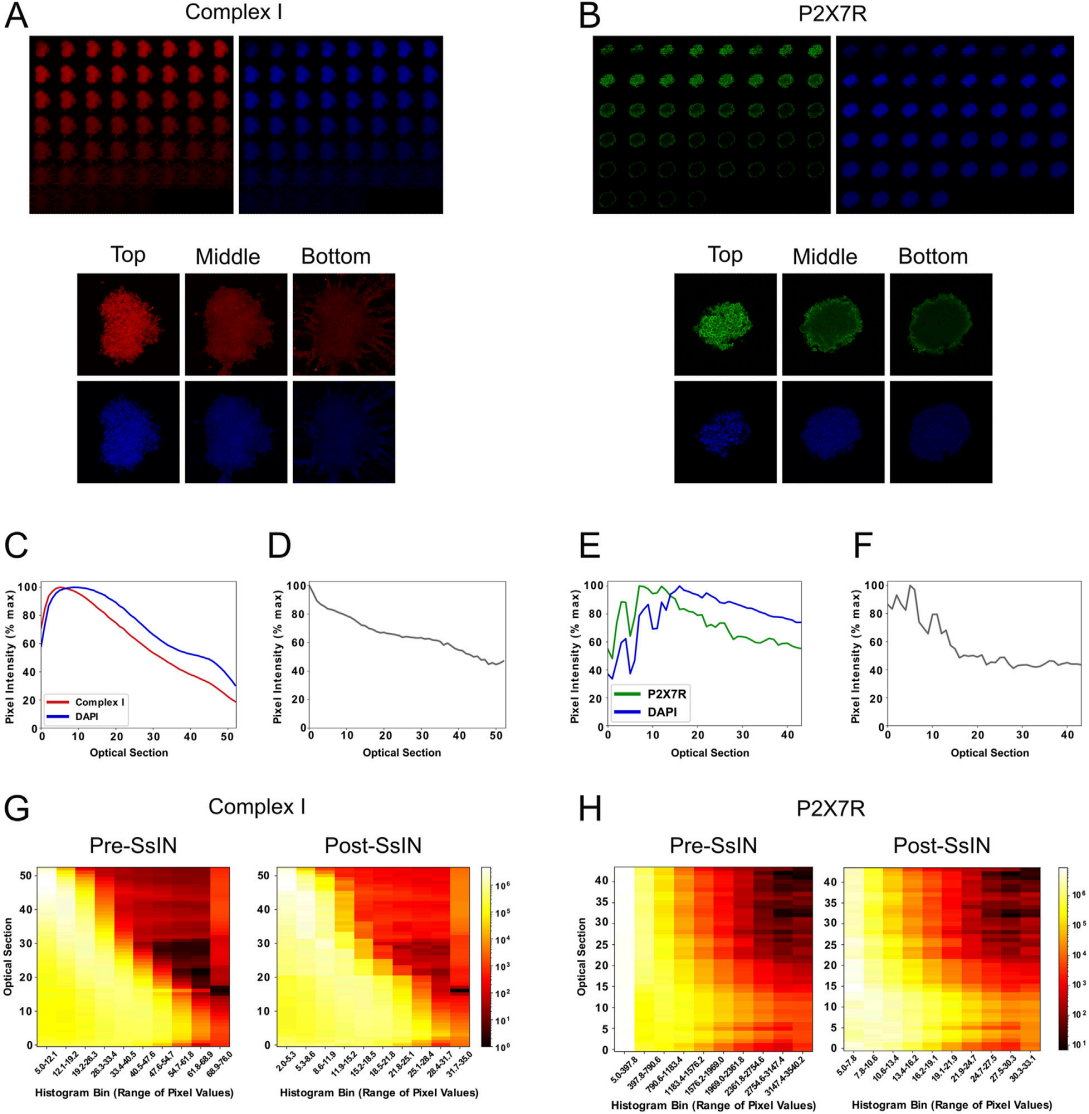
Normalization by SsIN Confirms Protein Localization Throughout Cell Clusters. Z-stack image sequence for 8-bit COXI (red) and DAPI (blue) along with reference images at the top, middle, and bottom of cell clusters **(A)**, and corresponding 16-bit Z-stack for P2X7R (green) and DAPI **(B)**. Average fluorescent signal collected at each optical section for COXI and DAPI **(C)** adjacent to its SsIN-normalized signal **(D)**. The same is shown for P2X7R **(E,F)**. A heatmap of histograms showing the distribution of SOI pixel intensity values at each optical slice pre- (left) and post- (right) SsIN for COXI **(G)** and P2X7R **(H)**.

As light passes through thick biological material such as tissue, varying refractive indexes cause light scattering and ultimately fluorescence quenching (Stanciu et al., 2010). As a proof of principle, we evaluated the effect of these phenomena on fluorescence intensity across the depth of 3D cell culture models. Human U87 cells were cultured until they formed cell clusters, followed by a staining protocol for DAPI (Ferro et al., 2017). Cell clusters ranged from ∼30 μm to 70 µm in thickness, near the penetration depth of confocal microscopy reported to be ∼100 µm (Graf and Boppart, 2010). DAPI binds to DNA stoichiometrically and its fluorescence possesses a linear relationship relative to DNA content (Ferro et al., 2017; Kubista et al., 1987). To confirm that light attenuation in Z-stacks was not exclusive to DAPI or a particular fluorescent probe, we immunostained cell clusters for the following SOIs simultaneously: cell membrane P2X7 receptor (P2X7R), a widely expressed protein in the central nervous system (Leeson et al., 2018), and mitochondrial complex I (COXI, NADH dehydrogenase 1 alpha subcomplex subunit 9). Focal plane brightness was visibly diminished in deeper planes for DAPI and each SOI (Figures 2A, B).

To quantitatively confirm depth-related fluorescence intensity loss, confocal images were exported as TIFF files and analyzed in ProDiVis. Excluding zero-value pixels when computing the section-specific mean (SSM) fluorescence intensity ensured quantification of fluorescence was not affected by large regions of the focal plane where no cells were present. Each graph (Figures 2C, E) contains parallel DAPI and SOI signals of all optical sections of the same cell cluster. The SSM whole-optical section analysis accounts for multinuclear cells or cells at various stages of the cell cycle by calculating the mean DAPI pixel intensity values. As expected, signal for DAPI decreased with imaging depth, with a decrease in fluorescence of up to 80% in deep focal planes *versus* shallow planes (Figures 2C, E). For each cell cluster, the highest signal for DAPI was detected at a depth range of 0–10 optical sections and proceeded to decrease beyond that point. Each optical section equates to a ∼1 micron step size per section, dependent on excitation and emission wavelengths while using a 1 AU pinhole. Similarly, intensity of the SOI was also depth dependent. The signal detected for P2X7R and COXI all had maximum signal intensity in the range of 0–10 optical sections. These observations suggest that if the different SOI had distinct spatial distribution within the 3D structure, the decrease in signal intensity as a function of depth shown in Figures 2C, E would prevent an accurate representation of the SOI 3D location.

### Section-specific-intensity-normalization ensures proportionality throughout imaging depth

The effects of SsIN on depth-related intensity loss were visualized by graphing the mean pixel value pre- and post- normalization. Although ProDiVis was not developed to produce pixel uniformity in Z-stacks, we still mitigated fluorescence intensity loss by about 20% for the cluster stained for COXI (Figure 2D). To show that normalization by SsIN does not markedly transform the distribution of pixel intensities per optical section, we generated multidimensional histograms for pre-and post- SsIN normalized Z-stacks. While the distribution of pixels per image in the Z-stack for COXI remained similar, after normalization, there was a considerable increase in the pixel values corresponding to optical sections 10–50, suggesting a more homogeneous distribution of the signal across its depth (Figure 2G). The mean pixel value and distribution of pixels for the cell cluster stained for P2X7R on the other hand, remained unchanged, suggesting that the distribution of the P2X7R signal may be indeed more restricted to the outer cellular layers of the cell cluster (Figures 2F, H). By adding SsIN to an image analysis pipeline, users can gain insight on the relative abundance of their signal of interest, ultimately improving the understanding of biological features without data removal.

### Section-specific-intensity-normalization accurately Reconstructs input images

**FIGURE 3:**
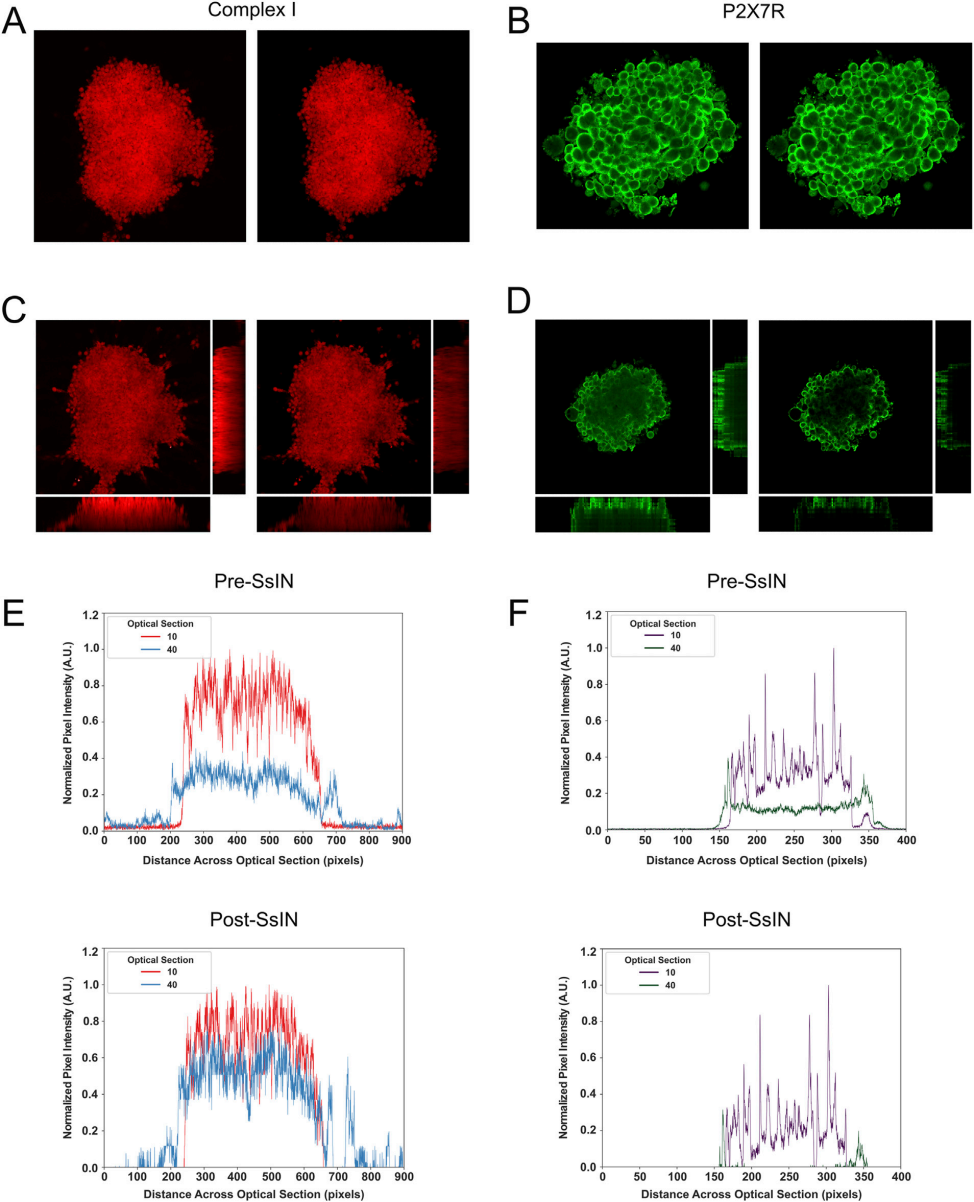
Images Are Reconstructed by SsIN with High Fidelity. Representative image pre- (left) and post- (right) SsIN labeled for COXI **(A)** or P2X7R **(B)**. Pre- (left) and post- (right) SsIN orthogonal projections for COXI **(C)** and P2X7R **(D)**. Fluorescence intensity profile across the diameter of cell clusters pre- (top) and post- (bottom) SsIN for COXI **(E)** or P2X7R **(F)**.

Another important SsIN feature is the ability to produce normalized images for use with existing images or image analysis software. To confirm that SsIN normalization techniques can accurately represent the localization of SOIs, we took SsIN output images and reconstructed original Z-stacks and orthogonal projections of cell clusters in ImageJ/Fiji (Figures 3A, B). For each SOI, input orthogonal projections all inherently biased fluorescence in favor of the upper optical sections even when signal was detected throughout cell clusters (Figures 3C, D). When we performed SsIN and reconstructed orthogonal images of their respective cell clusters, we accurately represented the localization of COXI and P2X7R when compared with the input images. Pixel intensity became more uniform in the Z-stack representing COXI signal, indicating its homogenous localization throughout cell clusters. However, P2X7R localization remained unchanged when comparing pre- and post- SsIN.

To further investigate the effect of SsIN in reconstructed images, we examined the fluorescence intensity profile across cell clusters. We analyzed optical sections 10, 40, corresponding to the upper and lower focal planes, respectively. The fluorescence profile revealed that COXI was homogenously distributed throughout the cell clusters, while P2X7R was mainly localized at the top and sides (Figures 3E, F). We also observed a decline in fluorescence intensity with depth, as anticipated. However, SsIN reconstructed images magnified the differences in fluorescence intensity not seen in input images. For instance, after SsIN, the fluorescence intensity of COXI in optical section 40 became comparable to optical section 10. In contrast, for P2X7R, SsIN reduced background noise and confirmed that P2X7R remained localized on the sides of cell clusters in optical section 40, while being uniformly distributed across the cell cluster in optical section 10. These results suggest that normalization with SsIN can reduce bias and accurately represent the distribution of labeled proteins in the 3D specimens.

### Section-normalized-intensity-projection provides a complementary method to represent 3D Z-Stacks

To create a simple and effective representation of signal distribution throughout Z-stacks, we developed SNIP–an average intensity projection of x, y, and z dimensions. The purpose of SNIP is to offer a projection method that emulates a commonly used technique: an orthogonal projection. SNIP does not modify the underlying biological features of a Z-stack, it enhances subtle differences seen within a specimen to facilitate further analysis.

**FIGURE 4:**
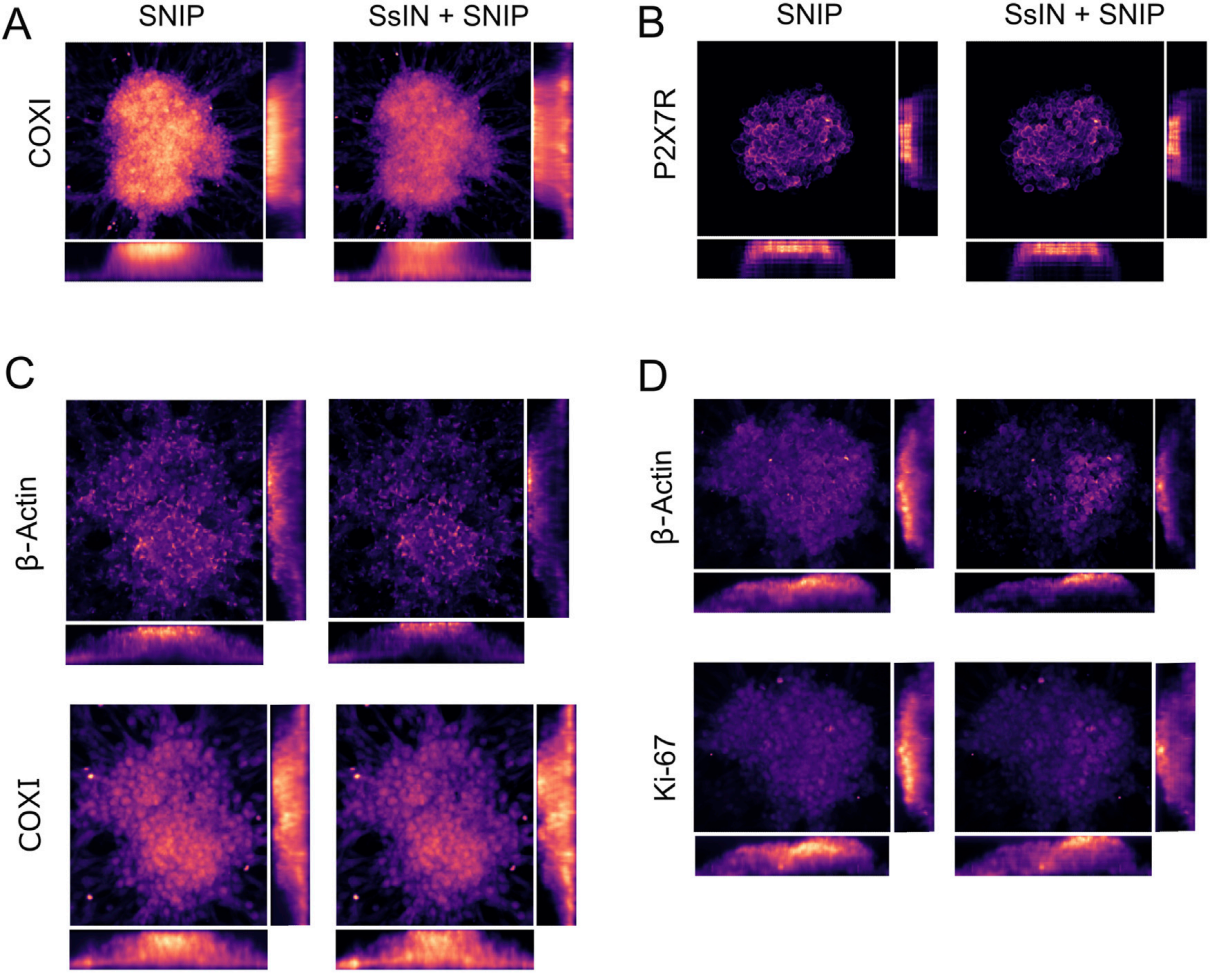
SNIP-Based Reconstruction Produces High Fidelity Heatmaps **(A–D)** Representative heatmaps of SNIP (left) and SsIN + SNIP (right) orthogonal views for COXI **(A)** and P2X7R **(B)**. Additional cell clusters were co-immunostained for β-Actin and COXI **(C)** and Ki-67 and β-Actin **(D)**.

To demonstrate the effectiveness of SNIP, we generated pre- and post-normalization SNIP heatmaps of cell clusters stained for COXI or P2X7R. With SNIP, we observed both SOIs were primarily localized in the upper half of cell clusters. However, this signal localization may not be representative of the biological context. For example, SsIN + SNIP confirmed that COXI signal was previously underrepresented at the bottom of cell clusters. After normalization, we observed homogenous distribution of COXI independent of depth (Figure 4A). P2X7R distribution on the other hand, remained at the top of cell clusters (Figure 4B). In addition, we co-immunostained cell clusters for COXI and β-Actin or Ki-67 and β-Actin. In both cases, β-Actin and Ki-67 were primarily localized at the top of cell clusters (Figures 4C, D). Before image processing, each fluorescence intensity decreased with imaging depth (Supplementary Figure S1).

In our particular experimental design, in some cases cells attached to the plate in a monolayer surrounding cell clusters interfered with SNIP representation of the clusters because the excitation and emitted light was not attenuated by any thick biological material. This was the case for cell clusters represented in Figures 4C, D. We circumvented this problem by cropping the original image to only include the cell clusters. Prior to image cropping, the distribution of COXI was unchanged when comparing its SNIP *versus* SsIN + SNIP heatmap (Supplementary Figure S2A). In cropped images, we were able to better dissect the differences in COXI and β-Actin localization. After removing the cells surrounding the cell cluster, the initial non-normalized SNIP projection showed a bias for fluorescence intensity at the top of cell clusters. However, COXI signal was equally represented throughout the cluster after normalization (Supplementary Figure S2B). For SOIs that have a depth-independent or dependent localization, SNIP was able to accurately represent pixels and highlight differences in biological features already present in the images. This analysis exemplifies the importance of the experimental context set by the user when applying these tools.

We further evaluated the accuracy of ProDiVis by using microscopy data from the public repository Image Data Resource (IDR) (https://idr.openmicroscopy.org/). The data included 8-bit Z-stacks from Esteban et al. (2022) corresponding to experiment Idr0124, atlas specimen E36, containing high-resolution confocal images with 654 optical sections (∼0.8 µm thick) used as part of a study that established a 3D description of early mouse heart development. This Z-stack represents a ∼5-fold increase in depth *versus* the cell clusters. We analyzed cells expressing a fluorescent probe (membrane GFP) to represent the mesoderm, counterstained with DAPI. We observed a similar loss of intensity as a function of imaging depth (Supplementary Figure S3A, C), and normalization by SsIN recovered pixel intensity loss (Supplementary Figure S3B, D). After normalization, SNIP heatmaps were able to accurately represent a frontal view of the mesodermal tissue (Supplementary Figure S3E, F) (Esteban et al., 2022).

## Discussion

Here, we developed ProDiVis, a Z-stack analysis and validation suite that proportionally visualizes signal distribution in 3D samples. To our knowledge, no existing Z-stack normalization technique involves normalization by a fluorescent housekeeping signal. ProDiVis does not alter the biological features within Z-stacks; rather, it accentuates differences that may have been hidden by the limitations of confocal microscopy. The straightforward nature of ProDiVis offers a valuable tool for researchers seeking to improve the visualization and analysis of Z-stack information without introducing potential artifacts.

Several analytical methods have previously been developed to address light attenuation and photobleaching in Z-stacks (Michálek et al., 2010; Čapek et al., 2006; Michálek et al., 2008; Yayon et al., 2018). One way to circumvent light attenuation is histogram warping, which creates a standard reference histogram based on the grayscale histogram of each image in a Z-stack. The histogram of each optical section is then matched to the reference histogram to achieve the best possible contrast and brightness (Čapek et al., 2006). Histogram warping assumes if multiple images of the same biological feature are taken, the brightness of each feature should be the same. This can be a useful method in image analysis, but not always optimal, particularly when analyzing biological data. Confocal optical sectioning often relies on collecting overlapping signals from each optical section, where each recorded optical section becomes an average of multiple overlapping scans. Therefore, an optical section may not always be viewed as an individual image but rather a combination of sections part of a larger 3D specimen. In a biological context, the distribution of a protein within a 3D specimen can vary both spatially and in a depth-dependent manner. Thus, a particular protein may not conform to the assumptions necessary for histogram warping, which we aimed to address with ProDiVis.

There are many open- and closed-source programs to view microscopy images in 3D space which contain integrated functions to correct fluorescence intensity loss. These include but are not limited to ImageJ/Fiji, Imaris, BioImageXD, 3D Slicer, Microscopy Image Browser, Image-IN, and Napari (Chiu and Clack, 2022; Gupta et al., 2022; Schneider et al., 2012; Fedorov et al., 2012; Belevich et al., 2016; Kankaanpää et al., 2012). Several of the mentioned 3D viewers have their own capabilities of correcting brightness and contrast issues in fluorescence microscopy images. For example, 3D Slicer, ImageJ/Fiji, and Napari, have a bleach correction function which uses multiple methods to correct fluorescence intensity (Miura, 2020). These methods include a simple ratio method–which assumes that the average observed intensity is constant throughout the imaging time (or depth, in our case). The exponential method fits intensity loss to an exponential curve that represents fluorescence decay. Finally, the histogram matching method, matches the histogram of each decayed image with a reference image histogram. While bleach correction is primarily used for time-lapse imaging, it can be used for Z-stacks as well. It is also common to find a gamma correction in 3D viewers mentioned above. While the human eye does not perceive luminance in a linear fashion, cameras and detectors do. Therefore, a 1X *versus* 2X signal may be detected on an instrument but that visual difference is not able to be noticed by the human eye. Gamma correction fits an exponential curve to the histogram of an image, resulting in non-linear scaling, where the distribution of pixel intensity values become distributed more evenly (Singnoo and Finlayson, 2010). Each viewer in 3D space we have tested indeed has gamma correction capabilities. Imaris on the other hand, has a dedicated feature called “Attenuation Correction” which is designed to maintain even illumination of structures through Z-stack depth, similar to gamma correction. This feature calculates a coefficient representing the rate of pixel loss per optical section which is then used to exponentially rescale each image. This requires the user to select an area at the top and the bottom of the Z-stack that are assumed to have the same intensities.

Intensify3D is a program that performs spatial and temporal correction to achieve illumination uniformity across x, y, and *z*-axes, allowing for the correction of fluorescent signals in large Z-stacks while also resolving small biological structures (Yayon et al., 2018). Intensify3D assumes a homogenous background intensity throughout a 3D structure to separate signal from background. It then uses background to perform normalization across the Z-stack. It is important to note that Intensify3D and ProDiVis have different goals and applications. ProDiVis is designed to visualize underlying differences in images without requiring complex preprocessing, while Intensify3D is designed to enhance and quantify biological features. As far as we know, Intensify3D is the only other normalization method for Z-stacks that circumvents the limitations associated with the fundamental depth limit and would provide complementary information to any analysis performed using ProDiVis.

A variety of chemical-based methods are also used to increase imaging depth. Optical clearing, for example, improves the ability to image thick samples by reducing refractive index changes, ultimately reducing light scattering (Brenna et al., 2022). Clearing tissue increases transparency, allowing greater light penetration. Despite a growing number of methods/protocols developed in recent years for tissue clearing, this method can present several drawbacks. For example, tissue clearing methods vary in their ability to increase transmittance. Such methods also induce size changes to the sample, reducing brain sections up to 35%. The clearing method may alter the retention of fluorescence and total imaging depth, which can range from 200 µm to ∼1200 µm (Wan et al., 2018). Optical clearing can also decrease emission light intensity of widely used fluorophores and increase crosstalk between emission spectra due to an emission peak shift (Eliat et al., 2022).

In an effort to maintain the biological features of each particular sample, ProDiVis provides an alternative approach to current complex solutions. ProDiVis is therefore a fast and minimally invasive method for image processing that requires no chemical alteration. ProDiVis does not introduce chemical or physical handling of the sample. Here, we used ProDiVis with fluorophores emitting in the visible spectrum, although ProDiVis is not limited to any particular dye or wavelength. For example, choosing an excitation/emission wavelength pair is important in deep imaging as longer wavelengths have greater penetration capacity but sacrifice spatial resolution (Helmchen and Denk, 2005; Sanderson et al., 2014). ProDiVis does not perform any pixel reassignment, but instead, standardizes pixel values across a reference value to increase proportionality across optical sections. For groups new to imaging thick samples, they can follow the classical fixation and antibody staining protocol, followed by Z-stack reconstruction where ProDiVis can be used.

As proof of principle for this study, we grew glioblastoma cell clusters and fixed and stained them without using optical clearing methods. Our samples were below the thickness that is traditionally needed for optical clearing; however, due to a combination of imaging time, light scattering, and photobleaching, we observed a substantial fluorescence intensity loss as a function of depth. By combining raw image data with normalized images acquired from ProDiVis, we provided an additional method to confirm that differential protein localization is not mutually exclusive to cell cluster depth. The spatial distribution of a given SOI may be dependent on intracellular location, cell type, relative size of the specimen, and the location of a particular cell within that specimen. P2X7R was expressed on the plasma membrane of cells located in the periphery of glioma cell clusters. Using ProDiVis, we confirmed that P2X7R expression in cell clusters was most abundant in the periphery of clusters and not an artifact of imaging methodology. In contrast, when analyzing the distribution of COXI, an analysis of the original Z-stack suggested increased levels of COXI present in cells located at the top of cell clusters. However, after applying SsIN + SNIP, the generated heatmap evidenced a mostly uniform COXI signal throughout.

## Conclusion

In conclusion, ProDiVis enhances the visualization of optical sections, with the goal of accentuating differences that are not readily visible. Through normalization with ProDiVis, we proportionally compared each optical section within a Z-stack and reconstructed normalized images with high fidelity. We hope this method improves the way biologists interpret and analyze their data by improving the contrast of images without compromising the biological meaning of the raw data. The SsIN + SNIP approach further refines this method by normalizing prior to heatmap generation, aiding in the visualization of protein distributions in 3D specimens.

## Limitations and future work

ProDiVis is a qualitative and complementary method to better dissect and validate what information is currently present. It does not increase resolution or decrease noise. The quality of normalization provided by ProDiVis ultimately depends on the user-selected normalization signal. ProDiVis assumes there is similar photon attenuation between the two channels. Depending on the fluorophores chosen, this may not always be the case, and normalization by ProDiVis could produce incorrect results. While we wanted to provide a simple, easy to use method, its limitation lies within selecting a good normalization signal relevant to the user’s work.

Since ProDiVis uses histogram thresholding, where the user selects a defined range of pixel values to be included in analysis–input images must have minimal background and experimental artifacts due to imaging. While very useful, histogram thresholding is a rather basic form of image segmentation. For example, our threshold is a fixed range of pixel values that is defined at the beginning of normalization and that range is applied to each image in the Z-stack. Under the assumption that pixel values decrease across imaging depth, lower pixel values deeper in the sample that represent true signal may be omitted from normalization. This may also play a factor in images or Z-stacks with uneven illumination. If a biological feature does not have a uniform pixel intensity, a similar problem may occur, where parts of it are removed because the pixel values are below the lower threshold limit.

## Data Availability

The datasets presented in this study can be found in online repositories. The names of the repository/repositories and accession number(s) can be found in the article/Supplementary Material.
