# Supplementary material for: ProDiVis: a method to normalize fluorescence signal localization in 3D specimens
Source: Front Cell Dev Biol. 2024 Sep 23;12:1420161. doi: 10.3389/fcell.2024.1420161 (PMC11456528; doi:10.3389/fcell.2024.1420161)
Supplement: Supplementary file 1 [file DataSheet1.pdf]

Supplementary Material

ProDiVis: A Method to Normalize Fluorescence Signal Localization in 3D Specimens

Kyle T. Nguyen[1, †], Alexandre R. Sathler[1, †], Alvaro G. Estevez[1, 2, 3], Isabelle E. Logan[1], and Maria Clara Franco[1, 2, 3 *]

[1]Department of Biochemistry and Biophysics, Oregon State University, Corvallis OR, USA

[2]Herbert Wertheim College of Medicine, Florida International University, Port St. Lucie, FL, USA

[3]Center for Translational Science, Florida International University, Port St. Lucie, FL, USA

[†]These authors contributed equally and share first authorship

*** Correspondence:**
Maria Clara Franco
marfranc@fiu.edu

Keywords: 3D specimen, heatmap, confocal microscopy, fluorescence microscopy, protein distribution, signal normalization, imaging, image analysis

This document includes:

Supplementary Figures 1 to 3

Data Availability Statement

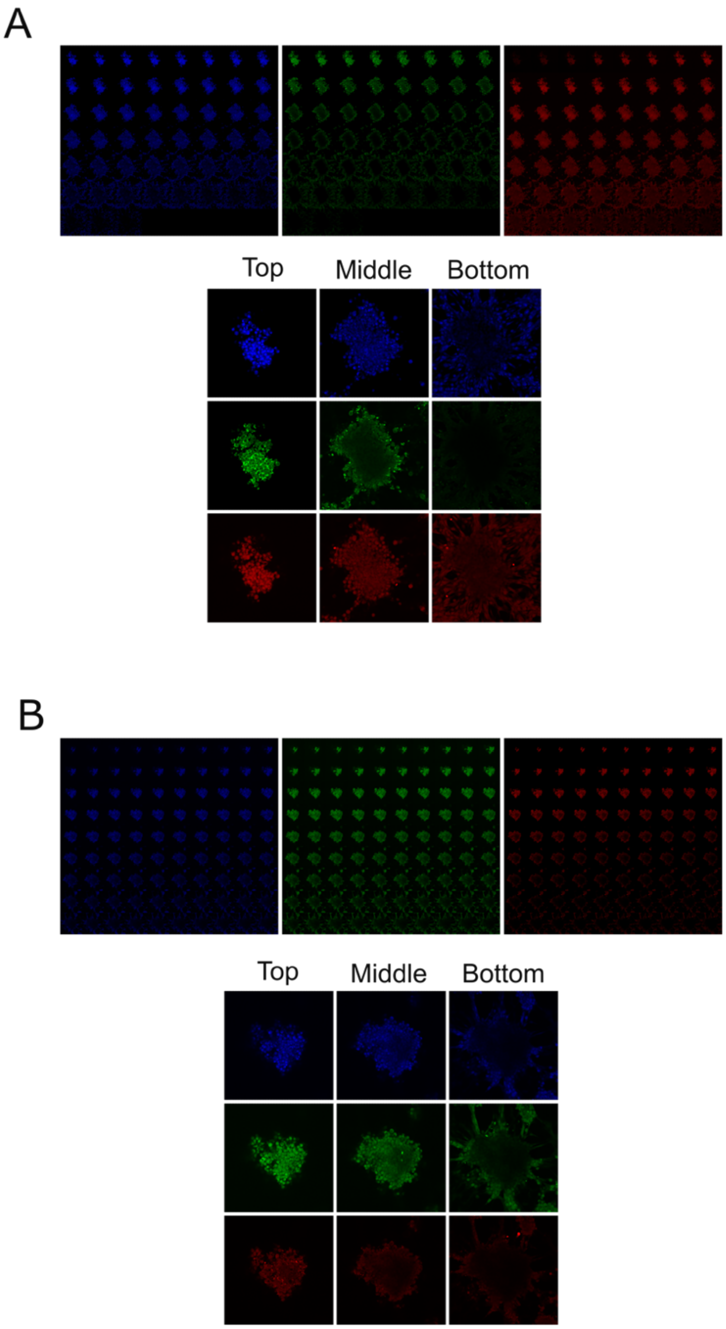

Supplementary Figure 1. (A) Cell cluster image sequence for DAPI (blue), β-Actin (green), and COXI (red) with reference images at the top, middle, and bottom of cell clusters. **(B)** Cell cluster image sequence for DAPI (blue), Ki-67 (green), and β-Actin (red).

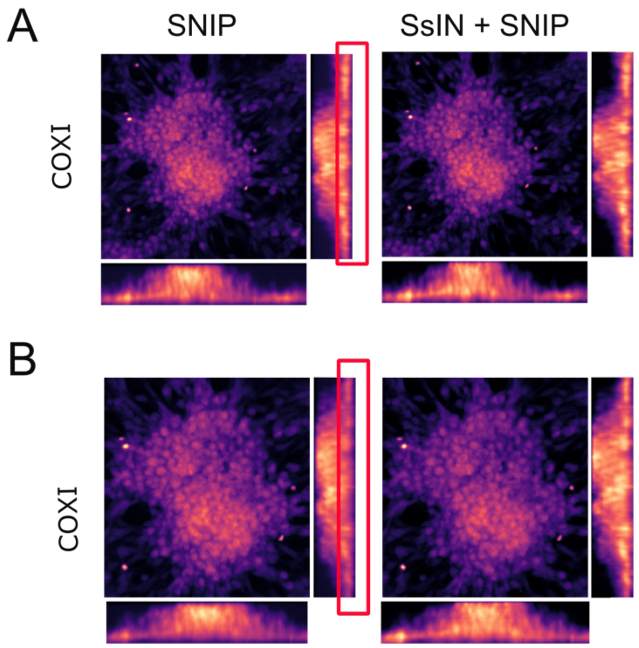

Supplementary Figure 2. (A) Heatmap for cell cluster stained for COXI using SNIP (left) or SsIN + SNIP (right) prior to image crop. **(B)** After cropping the image, the heatmaps are shown again with SNIP (left) or SsIN + SNIP (right). Red boxes indicate the region in depth where more fluorescence intensity was previously observed from surrounding monolayer cells prior to cropping.

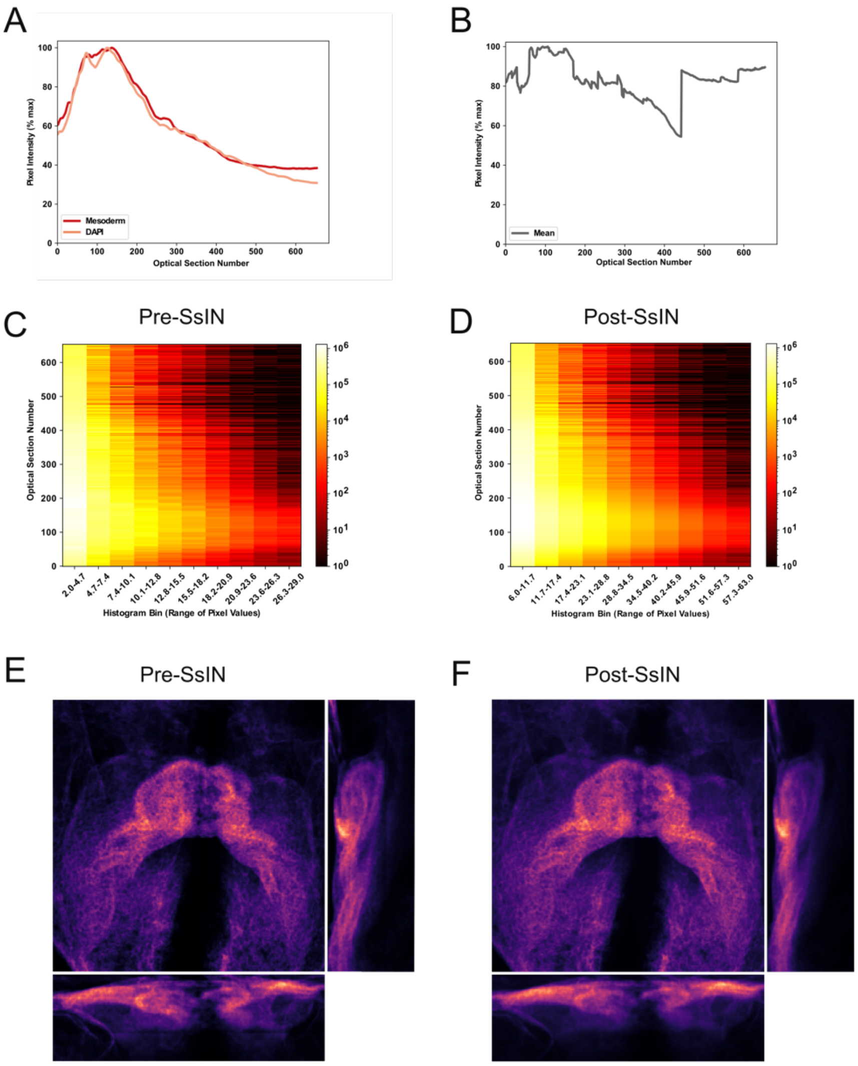

Supplementary Figure 3. Average fluorescent signal collected at each optical section for mesoderm (GFP signal), and DAPI **(A)** adjacent to its SsIN-normalized signal **(B)**. Heatmap of histograms showing the distribution of SOI pixel intensity values at each optical section pre- (**C**) and post- (**D**) SsIN. Heatmaps of SNIP orthogonal views pre- (**E**) and post (**F**) SsIN for mesoderm normalized to DAPI signal.

Data Availability Statement

The original contributions presented in the study are included in the article/supplementary material, further inquiries can be directed to the corresponding author/s. The software generated for this study can be found in the repository: [https://github.com/FrancoLaboratory/ProDiVis]. Additionally, [https://github.com/FrancoLaboratory/ProDiVis-Images] provides access to confocal images to test the software.
